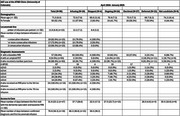# Treatment Characteristics With Lecanemab at the University of Colorado's Advanced Therapy in Neurodegenerative Disorders (ATND) Clinic

**DOI:** 10.1002/alz70858_106083

**Published:** 2025-12-26

**Authors:** Tara C Carlisle, Kavita V Nair, Stefan H Sillau, Lizel L Giron, Jennifer Kwak, Jody Tanabe, Samantha K. Holden, Victoria S Pelak

**Affiliations:** ^1^ University of Colorado Anschutz Medical Campus, Aurora, CO, USA; ^2^ Department of Neurology, and Department of Clinical Pharmacy, University of Colorado, Aurora, CO, USA; ^3^ University of Colorado Department of Neurology and Alzheimer's and Cognition Center, Aurora, CO, USA; ^4^ university of colorado anschutz medical campus, aURORA, CO, USA; ^5^ University of Colorado School of Medicine, Aurora, CO, USA

## Abstract

**Background:**

Clinical trials for FDA‐approved anti‐amyloid therapies (AATs) do not mirror the real‐world experience of patients seen by dementia specialists. The unique Advanced Therapy in Neurodegenerative Disorders (ATND) Clinic at the University of Colorado staffed primarily by one dementia specialist shares its experience.

**Objectives:**

To share the experience of the unique Advanced Therapy in Neurodegenerative Disorders (ATND) Clinic at the University of Colorado including characteristics and utilization of anti‐amyloid therapies (lecanemab) between April 2024 ‐ January 2025.

**Methods:**

Baseline characteristics including demographics, diagnostic assessments, lecanemab utilization, and Amyloid Related Imaging Abnormalities (ARIA) assessments were collected from ATND Clinic electronic medical records.

**Results:**

Patients were referred from the larger Memory Disorders Clinic at the University of Colorado, previously diagnosed with probable Alzheimer's disease, seen between April 2024 to January 2025 (*n* = 96) and not clearly meeting exclusion criteria for lecanemab. The current cohort includes those: in active diagnostic work‐up, 25.0% (*n* = 24); currently being infused, 19.8% (*n* = 19); who declined treatment, 28.1% (*n* = 27), were determined ineligible, 9.4% (*n* = 9), who deferred treatment, 13.5% (*n* = 13); stopped/transferred treatment (as of January 2025), 4.2% (*n* = 4). Mean age was 71.5 (+/‐8.0) years, with 52.1% women. Over half (59.4%) received a baseline MRI (undergoing the diagnostic work‐up) and the majority (88.9%) underwent a lumbar puncture compared to 9.5% who had an amyloid positron emission tomography scan to establish biomarker confirmation of Alzheimer's. About a fifth (20.9%) with *Apolipoprotein E* (*APOE4)* data tested homozygous for the APOEe4 allele. Two patients experienced ARIA while receiving lecanemab. On average, infusing patients completed 12.2 (+/‐7.2) infusions during the 10‐month observation period. The mean number of days between the first consultation with the dementia specialist in the ATND Clinic and the baseline MRI was 31.6 (+/‐23.1) (*n* = 47) days, and the first lecanemab infusion was 79.9 (+/‐38.3) (*n* = 19) days. Patients declined AAT treatment because of homozygous *APOEe4* status (37.0%) followed by concerns regarding modest efficacy (25.9%) and burden of treatment including cost (14.8%).

**Conclusion:**

The ATND Clinic's experience in the first year of treatment provides real world data of AAT experience. Investment in hiring more dementia specialists can increase patient capacity for AAT evaluation and management.